# Impact of Processing Technology on Macro- and Micronutrient Profile of Protein-Enriched Products from Fish Backbones

**DOI:** 10.3390/foods10050950

**Published:** 2021-04-27

**Authors:** Mehdi Abdollahi, Haizhou Wu, Ingrid Undeland

**Affiliations:** Department of Biology and Biological Engineering—Food and Nutrition Science, Chalmers University of Technology, SE 412 96 Gothenburg, Sweden; haizhou@chalmers.se (H.W.); undeland@chalmers.se (I.U.)

**Keywords:** seafood, nutritional value, by-products, pH-shift method, mechanical separation

## Abstract

Impacts of processing technology (mechanical separation and pH-shift processing) on protein recovery from salmon, herring and cod backbones and the content of macro- and micronutrients in the recovered protein enriched products were investigated. Mechanical separation led to higher protein recovery compared with the pH-shift process and using both techniques, recovery ranked the species as herring > salmon > cod. However, the pH-shift process up-concentrated protein from herring and salmon backbones more efficiently than mechanical separation by removing more fat and ash. This consequently reduced n-3 PUFA and vitamin D content in their protein isolates compared with the backbones and mechanically separated meat (MSM). Cod protein isolate, however, contained higher levels of these nutrients compared with MSM. Mechanical separation concentrated vitamins E and C in salmon MSM but not for cod and herring. Opposite, pH-shift processing reduced levels of these two vitamins for cod and herring backbones, while vitamins D and C were reduced for salmon. For minerals, selenium, calcium, magnesium, and potassium were lower in protein isolates than MSM, while copper, zinc, iron and manganese were similar or higher. Overall, there is a major potential for upcycling of fish backbones to food ingredients, but processing technology should be carefully balanced against the desired nutrient profile and final application area.

## 1. Introduction

Seafood are key components of a healthy human diet which provide a unique combination of high-quality proteins and amino acids, long-chain n-3 polyunsaturated fatty acids (LC n-3 PUFAs), vitamin D and minerals e.g., selenium. Documented health benefits of seafood along with the growing world population have steadily increased the demand for this food commodity. However, wild fisheries cannot be expanded further, and sustainability issues have inevitably arisen within the supply and use of marine resources, also the farmed ones [1]. A typical example is the increasing demand for pure fillets among consumers, something which results in losses of 40–60% of the initial biomass from the food chain in the form of filleting coproducts [1]. Some of these coproducts, particularly the backbone which carries a significant amount of residual fillet, are very good sources of high-value nutrients like proteins, LC n-3 PUFA, vitamin D and other micronutrients [2], therefore deserving a better destiny than e.g., fish or mink feed. However, the sensitive nature, along with the complex bony structure call for gentle valorization technologies which can maintain the targeted nutrients and product-forming capacity intact. 

Mechanical meat–bone separation is a well-established technology that has been used in the poultry industry for decades [3]. Low investment cost, simplicity of operation and high separation efficiency have quickly expanded interest in its application for upcycling of fish processing coproducts e.g., trimmings and backbone to food [4]. In this technique, residual muscle on the by-products is removed from bone by applying low (<10^4^ Pa) or high (>10^4^ Pa) levels of pressure [5]. This is mainly achieved by passing the coproducts through a nip between a perforated steel drum rotating against a rubber belt which counter-rotates [6]. However, the used pressure may cause loss of endogenous muscle structure and also exposes muscle components to pro-oxidative heme pigments and enzymes which are released during the separation. The latter could negatively affect the nutrient stability and sensorial properties of the mechanically separated meat (MSM) limiting its final application [7]. Given the large differences in morphology between backbones from different species, and their different levels of pro-oxidants such as hemoglobin (Hb) as well as fat and connective tissue, the nutritional value of MSM is expected to be highly dependent on the type of input raw material. Although a few studies exist on MSM-production from beheaded fish [5,8], there are no publications comparing MSM from fish backbones alone, and also not from several species side by side.

Another process that has shown good potential for the valorization of fish processing coproducts is the so-called pH-shift method. The process involves selectively extracting proteins from homogenized coproducts using a high (>10.5) or a low (<3.5) pH, followed by precipitation at their isoelectric point (pI). The process has been widely investigated as a tool to produce food-grade high-quality gel-forming proteins, and most recently also fish oil, from fish processing coproducts [9]. The process can effectively concentrate proteins of fish processing coproducts by removing the bone residue and some of the fat resulting in a protein paste with good levels of essential amino acids and good functional properties [10,11]. However, based on the addition of significant amounts of water, part of the nutrients in the used raw material might be unintentionally removed by cofloatation or coprecipitation during the first centrifugation step and/or by solubilization into the water phase which is partly removed during the dewatering step. Furthermore, compared with mechanical separation, the pH-shift process is a more complicated technology requiring higher levels of processing and investment. Despite the large numbers of studies testing the pH-shift process for protein recovery from different marine resources [12,13], the effects on the micronutrient composition of the final protein enriched product when using different input raw materials have not been systematically elucidated, and also not compared with MSM. 

The present study therefore aimed to investigate the effects of mechanical separation vs. pH-shift processing on protein recovery as well as content of macro- and micronutrients in the recovered protein enriched product using backbones of cod, salmon and herring. The impacts of the two valorization technologies on the contents of protein, fat, amino acids, fatty acids, vitamins and minerals in the protein-enriched products recovered from backbones of the three fish species were in focus. 

## 2. Materials and Methods

### 2.1. Fish Backbone Samples

Fresh backbones of salmon (*Salmo salar*) (in total 30 kg) and cod (*Gadus morhua*) (in total 25 kg) were provided by Fisk Idag AB (Gothenburg, Sweden) while herring backbones (in total 40 kg) were provided by *Scandic Pelagic AB* (Ellös, Sweden). The backbones were covered with ice-filled plastic bags immediately after processing of the fish and divided into two batches. One smaller batch (10 kg) was transported to the marine lab at Chalmers University of Technology within 3 h post-processing. Upon arrival, backbones were grinded in a tabletop mincer equipped with a hole plate having 4.5 mm holes (C/E22 N, Minerva Omega Group, Bologna, Italy) and the mince was directly used for pH-shift processing. The second batch (15–20 kg) was transported to Fisk Idag AB (Öckerö, Sweden) and subjected to mechanical separation as described below. 

### 2.2. pH-Shift Process and Mechanical Separation

The minced backbones from salmon, cod and herring were separately subjected to pH-shift processing according to the method described by Abdollahi and Undeland [14]. First, 800 g of each minced backbone was mixed with 4800 mL of cold distilled H_2_O and the mixture was homogenized with a Polytron homogenizer (IKA, Staufen im Breisgau, Germany) at 25,000 rpm for 3 min. To facilitate separation of soluble proteins, lipids and collagenous components in the backbones, the pH of the homogenate was adjusted to 11.5 by adding 2 M NaOH, respectively. This was performed automatically using a titrator (907 Titrando, Metrohn AG, Zurich, Switzerland), where the pH was monitored by a Hamilton double pore electrode (Bonaduz, Switzerland). The homogenate was then incubated for 10 min on ice, followed by centrifugation at 8000× *g*, at 4 °C for 20 min. The centrifugation generated three layers including a floating emulsion layer containing the oil, a mid-layer containing solubilized proteins and a pellet made of collagenous residue and cellular membranes. The mid-layer was separated from the supernatant using a metal sieve and its pH was readjusted to 5.5 by adding 2 M HCL. After 10 min incubation on ice, the sample was dewatered by centrifugation at 8000× *g*, at 4 °C for 20 min. The moisture content of the protein pellet was further reduced to 80% with an extra cycle of centrifugation (10 min at 8000× *g*) and its pH was readjusted to 7 using 2 M NaOH [14]. 

For mechanical separation, 15–20 kg of backbones from each species was separately passed through a Baader 600 meat-bone separator (Baader 600, Baader Fish Processing Machinery, Germany). The remaining muscle on the herring and salmon/cod backbones was hereby separated by squeezing them through a perforated drum with 3 and 5 mm hole diameters, respectively. The MSM was immediately collected, packed in plastic zip lock bags and frozen at −80 °C until further studies. The trials for mechanical separation were done at least on two different batches of backbones for each fish species, each batch being 15–20 kg. 

### 2.3. Protein Recovery Measurement

Protein recovery using mechanical separation and pH-shift processing for the three studied species was calculated by dividing total protein content of the MSM or protein isolates with the total amount of protein in the initial backbones, according to Equation (1).

(1) $$\mathrm{Protein}\;\mathrm{recovery}\;(\%)=\frac{Protein\;content\;in\;separated\;meat\;or\;protein\;isolate\times weight\;of\;recovered\;meat\;or\;isolate}{Protein\;content\;in\;fish\;backbones\times weight\;of\;processed\;backbones}\times 100$$

### 2.4. Compositional Analyses

The total protein content of initial backbones and their corresponding MSM as well as protein isolates was measured using a LECO nitrogen analyzer (TruMac-N, LECO Corp., St. Joseph, MI, USA) according to the Dumas method. A nitrogen-to-protein conversion factor of 5.58 was used to calculate the protein content of all fish backbones and protein isolates [15]. 

The total lipid content of the samples was analyzed using the method of Lee et al. [16], as modified by Undeland et al. [17]. The moisture content of the samples was measured by overnight heating at 105 °C. Ash content was also determined gravimetrically by heating the samples at 550 °C in a furnace for 6 h. Protein, fat and ash content are presented based on g/100 g of dry weight. 

### 2.5. Amino Acid Analysis 

Amino acid composition of backbones and their corresponding MSM as well as protein isolates of the three species was analyzed according to the method explained by Abdollahi et al. [18] and according to method developed by Özcan and Şenyuva [19]. Briefly, the samples were freeze-dried and ground to a fine powder. Then, 15–20 mg of each powder was weighted in screw cap glass tubes and subjected to acid hydrolysis by adding 4 mL of 6 M HCl and heating for 24 h at 110 °C. The samples were diluted with 0.1 N HCL, loaded in glass tubes and automatically run in an LC/MS (Agilent 1100 HPLC, Waldbron, Germany) equipped with a Phenomenex column (C18 (2) 250 μm × 4.6 μm × 3 μm), coupled to an Agilent 6120 quadrupole in the SIM positive mode (Agilent Technologies, Boeblingen, Germany). Collected data were then compared against previously run amino acid standards.

### 2.6. Fatty Acid Composition Measurement

Lipids were extracted from the backbones, their corresponding MSM and protein isolates according to Lee et al. [16] with some modifications as explained by Tullberg et al. [20]. Then, the lipid samples were methylated by following the method described by Cavonius et al. [21] which was based on methanolic HCL transesterification. After methylation, the toluene was evaporated under nitrogen gas and the fatty acid methyl esters were resolubilized by adding 1.0 mL of isooctane. The samples were then diluted in iso-octane and subjected to GC–MS analysis using an Agilent 7890 A GC system (Agilent Technologies, Santa Clara, CA, USA) equipped with a VF-wax column and interfaced with an Agilent 5975C triple-axis mass spectrometric (MS) detector in electron impact mode. Injection volume was 1 µL with a 15:1 split at an inlet temperature of 275 °C. C17:0 (Sigma-Aldrich, Heidelberg, Germany) was used as an internal standard and was added to the extracted lipids before their methylation. Data for each fatty acid are expressed as g/100 g of wet raw material.

### 2.7. Measurement of Vitamins D, E and C

Content of vitamin D in the initial backbones and the corresponding MSMs as well as protein isolates of the three species were determined according to the method explained by Standal et al. [22]. In brief, 0.4 g of homogenized and lyophilized fish sample was mixed with 1 g KOH, 5 mL ethanol:methanol (50/50 *v*/*v*) with 0.5% (*w*/*v*) pyrogallol, and internal standard D6–25(OH)D3 (Sigma Aldrich H-074), blanketed with N_2_ gas, sealed, and shaken in ambient temperature overnight. Then, 5 mL toluene was added, and the sample was treated for an additional 30 min; 2 mL H_2_O was added, and the upper organic phase transferred to a new test tube. The sample was extracted twice with 2 mL petroleum ether:diethyl ether (80:20 *v*/*v*). The removed organic phases were pooled, evaporated to a volume of approximately 1 mL, and washed with H_2_O until neutral pH was obtained. The organic phase was evaporated and dissolved in 2 mL 1% 2-propanol in heptane. The extracts were then subjected to solid-phase extraction (TELOS Silica, Kinetics, St Neots, Cambridgeshire, UK) according to the method by Jäpelt et al. [23]. Measurement of vitamin D3 was done by injecting the samples to an HPLC–MS (Agilent 1200 series system with an Agilent 6120 MSD single quadrupole, Agilent Technologies, Santa Clara, CA, USA). The samples were separated on two adjacent C18 columns (Luna, 250 mm, 3 µm C18(2) 100 A, Phenomenex, Torrance, CA, USA) by gradient elution with mobile phase A, 98% MeOH and B, Tetrahydrofuran/isopropanol/MeOH (30/30/40%). The gradient started at 100% A for 20 min then 100% B for 15 min, followed by 100% A, total runtime 50 min. Quantification of D3 was made against an external standard of Vitamin D3 (Alfa Aesar B22524). Results are expressed as µg/g dry w (*n* = 3).

α-Tocopherol content, i.e., vitamin E, of the samples was measured according to Larsson et al. [24] using HPLC. Briefly, 1 mL chloroform phase extract obtained using the Blight and Dyer method [25] was evaporated to dryness under N_2_, diluted in 0.2 mL methanol and centrifuged at 2000× *g* for 3 min. Then, 200 µL of the supernatant was transferred to vials for HPLC analysis. The chromatographic separation of tocopherol isomers was performed on a C18 column (Kromasil, 150 mm × 2.1 mm, 5 µm) using 98% methanol as mobile phase under isocratic conditions at a flow rate of 0.4 mL/min, and 5 µL injection volumes. The detection was performed with a fluorescence detector (Shimadzu RF-551, Kyoto, Japan) using 295 and 330 nm as excitation and emission wavelength, respectively. The concentration was quantified using an external α-tocopherol standard.

The content of ascorbic acid, i.e., vitamin C, of the backbones, MSMs and protein isolates was measured according to the method described by Lykkesfeldt et al. [26], with some modifications. Briefly, the fish samples were mixed 1:1 with meta-phosphoric acid (10% *w*/*v*) and disodium EDTA (2 mM) and vortexed. Before analysis, the samples were diluted 1:1 with Tris (2-carbozyethyl) phosphine (TCEP, 0.312 mM) in 1:10 McIlvaine buffer (0.46 M Na2HPO_4_, 0.27 M citric acid, pH 4.5) and 9:10 phosphate buffer (50 mM, disodium EDTA 20% (*w*/*v*), pH 2.8). Then, measurement of ascorbic acid in the prepared samples was done using a Jasco Corporation HPLC system (Tokyo, Japan), including an autosampler (AS-2057), a plus pump (AS-2080) and an Aquasil C18 analytical column (4.6 × 150 mm, Thermo Scientific, Sunnyvale, Wilmington, NC, USA). 

### 2.8. Measurement of Minerals

To measure minerals in the backbones, MSM and protein isolates, 0.25 g of each sample was subjected to acidic microwave digestion using milestone microwave laboratory system (Ethos Plus and a laboratory terminal 800 controller, Sorisole, Italy) equipped with MPR-300/12S (medium-pressure segmented rotor). The digestion was conducted in Teflon tubes in the presence of 3 mL of milliQ H2O, 0.75 mL of concentrated HNO_3_, and 0.15 mL of concentrated HCl at 180 °C for 20 min. The digested samples were diluted properly and used for measurement of Na, K, Ca, Se, Mg, Mn, Zn, Fe and Cu using flame atomic absorption spectroscopy with an Agilent 240/280 series AA spectrometers (Agilent Technologies, Palo Alto, CA, USA). Heme iron content was measured using 1 g of freeze-dried powder of each sample by measuring total heme using the acetone-based method of Hornsey [27]. The heme-iron content was then calculated with the factor of 0.0882 μg iron/μg heme.

### 2.9. Statistical Analysis

Statistical analysis was performed using SPSS software (IBM SPSS Statistics Version 24, IBM Inc., Chicago, IL, USA). One-way analysis of variance (ANOVA) was carried out to determine significant differences between sample groups, followed by Duncan’s multiple range test. Significance level was set at 0.05, below which the differences were considered significant.

## 3. Results and Discussion

### 3.1. Protein Recovery from Fish Backbones

**Figure 1:**
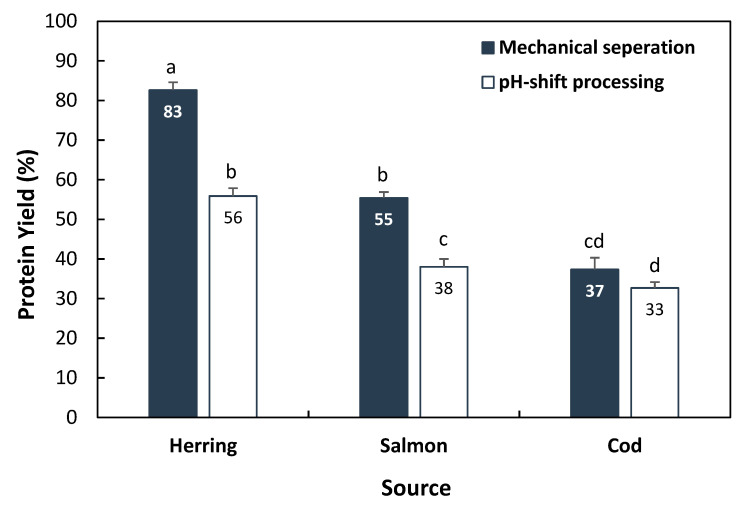
Protein yield using mechanical separation and pH-shift processing for recovery of protein-enriched products from herring, salmon and cod backbones. Results are shown as mean ± SD (*n* = 2). Different small letters show significant differences (*p* ≤ 0.05).

Protein recovery from backbones of the three studied species using pH-shift processing and mechanical separation is summarized in Figure 1. As can be seen, the highest protein recovery using both techniques was provided by herring backbone (55 vs. 83%) followed by salmon (38 vs. 55%) and cod (33 vs. 37%), reflecting that there was a higher ratio of muscle residue remaining on herring backbone compared to the two other species. This order was slightly different from what we recently found [14] when applying the pH-shift process on head + backbone of these three species; salmon > herring > cod, reflecting probably the relatively larger content of muscle in salmon compared to herring heads. The mechanical separation resulted in higher protein recovery (37–83%) compared with the pH-shift process (33–56%) for the three studied species, with the largest difference between the techniques found for herring (27%, *p* < 0.05), followed by salmon (17%, *p* < 0.05) and cod (only 4%, *p* > 0.05). Differences could be related to different structure and composition of the muscle residue and the amount of soft collagenous residue on the backbone of each species. While mechanical separation recovers part of the soft collagenous residues such as connective tissues and skin, these residues are effectively removed using the pH-shift process due to its high selectivity toward non-collagenous proteins. A weight yield of 40–53% was reported when mechanical separation was applied to beheaded rainbow trout (*Oncorhynchus mykiss*), sea bass (*Lateolabrax japonicus*) and sea bream (*Sparus aurata*) [28]. 

### 3.2. Macronutrients of the Recovered Protein-Enriched Products

Both mechanical separation and pH-shift processing concentrated protein from backbones (Table 1). Average protein content on dry weight (DW) basis in the original backbones of the three species (34–60%) increased up to 58–81% in the MSM and to 72–85% in pH-shift protein isolates. However, the protein concentration efficiency of both processes was highly species dependent. Previously, also Borgogno et al. [28] showed that fish species can affect the protein concentrating factor during mechanical separation. The authors found that MSM of beheaded seabass had a significantly (*p* < 0.05) lower content of protein than its fillet while beheaded sea bass and rainbow trout resulted in MSM with protein content equal with their fillets. The pH-shift process was significantly (*p* < 0.05) more effective compared with mechanical separation in up-concentrating protein when applied on herring and salmon backbone. This is mainly related to the higher efficiency of the pH-shift process in removing fat which becomes evident with high-fat input raw material. On the other hand, when cod, which is leaner, was used as input material, both processes resulted in a similar increase in protein content. Protein concentrating factor for meat-bone separation and pH-shift processing was highest for cod and salmon, respectively. Similarly, the pH-shift process gave rise to highly concentrated protein isolates (up to 80–85%, DW basis) when applied on different bony fish processing coproducts e.g., fish frame, head [29] and their mixture [14]. 

Salmon MSM showed significantly (*p* < 0.05) lower fat content (43% of dw) compared with its original backbones (52% of dw) while MSM from herring and cod backbones did not differ significantly from their corresponding input material (Table 1). Obviously, the distribution of fat in bone marrow vs. muscle will dictate how much fat can be removed together with the bone residue in mechanic separation. The results from salmon imply that this species, where ≥50% bones were removed, has a relatively large amount of high fat bone marrow or other bone-derived lipid deposits compared to cod and herring. However, for herring backbones, where the relative amount of bone was very small (Figure 1), the role of bone marrow is expected to play a minor role for the total MSM fat content, explaining why the comparison between salmon and cod backbones is more relevant. Borgogno et al. [28] found significantly lower fat content in MSM from beheaded sea bream compared with its fillet while there was no such differences in the fat content of MSM vs. fillets for sea bass and rainbow trout. 

Salmon and herring pH-shift protein isolates showed substantially lower fat content compared with their original backbones and their corresponding MSM. This is mainly related to the partitioning of neutral storage lipids into the floating emulsion layer emerging after the first centrifugation when using high-fat raw material as salmon and herring backbones. We recently showed that as much as 50–60% of the total fat was removed after the first centrifugation step as floating emulsion layer during pH-shift processing of herring and salmon heads plus backbones [9]. However, if the centrifugation force is high enough, a large portion of the phospholipids can also be removed into the first sediment, as membranes have a relatively high density. In addition, a certain part of the phospholipids may also be removed into the second supernatant formed during dewatering of the precipitated proteins [30]. Contrary, there was slightly, but yet significantly (*p* < 0.05), higher content of fat in cod protein isolate compared with its backbones explained by the more pronounced removal of collagenous residues as bones, skin and connective tissue, compared to the sedimentation or solubilization of membranes, resulting in an up-concentration of both fat and protein in the final isolate. This is in agreement with previous reports comprising pH-shift processing of lean fish raw materials, including cod head plus backbone [14].

Both MSM and protein isolates of the three species showed substantially lower content of ash compared with the initial backbones, which shows the high efficiency of both processes in removing bony residues (Table 1). However, in all the three cases, protein isolates had significantly (*p* < 0.05) lower amount of ash (2.18–2.51%) compared with their corresponding MSM (3.70–5.49%) revealing higher efficiency of the pH-shift process in removing bones and minerals compared with the mechanical separation. The lower protein purity in the MSM samples supports the hypothesis that the higher total protein yield obtained using the mechanical separation goes hand in hand with a less protein-enriched product. 

Overall, application of the pH-shift process for the valorization of herring and salmon backbone resulted in much more efficient purification and concentration of proteins via more efficient removal of fat and ash compared with the mechanical separation. In the case of cod, both processes acted similarly in terms of up-concentration of protein and fat, but the pH-shift process more effectively removed ash. 

### 3.3. Amino Acid Composition

Both MSM and protein isolates of the three species had lower content of nonessential amino acids than the backbones, especially proline and glycine (Table 2) which are enriched in the collagenous residues [31] being removed in mechanical separation and pH-shift processing. These findings agreed with our previous studies where we subjected mixed fish processing coproducts to pH-shift processing [11,31]. For herring, MSM and protein isolates also had reduced content of some other nonessential amino acids including arginine, tyrosine, aspartic acid and glutamic acid compared with its backbones. For the protein isolates, this resulted in a significant (*p* < 0.05) enrichment of some essential amino acids (EAA) including leucine and isoleucine, compared with the herring and salmon MSM and backbone raw material. For cod, the content of these EAA ranked the samples as protein isolates > MSM > backbone (*p* > 0.05). Both the total content of EAA, and the EAA to total AA ratio, was higher in protein isolates of the three species compared with their MSM and backbones (*p* < 0.05) reflecting the efficient up-concentration of the EAA-rich myofibrillar and sarcoplasmic proteins during pH-shift processing. This is in line with the results of Chen et al. [10,32] and Taskaya et al. [33] who found higher content of EAA in protein isolates from trout and carp by-products compared with their original by-products. Overall, the EAA-content of protein isolates and MSM was well above recommendations by FAO/WHO for adults, except for the content of leucine in the MSM which was slightly lower than the recommendations by FAO/WHO for adults [34]. However, the content of valine, leucine and phenylalanine in both MSM and protein isolate of herring and cod did not meet the recommendations by FAO/WHO for infants. For samples from salmon, it was only the content of phenylalanine which was lower than the recommendation by FAO/WHO for infants [34].

### 3.4. Fatty Acid Composition 

Our results reveled that there were no significant differences (*p* > 0.05) in the content of total saturated fatty acids (SFA), monounsaturated fatty acids (MUFA), polyunsaturated fatty acids (PUFA), n-3 PUFA and LC n-3 PUFA between herring MSM and its original backbone (Table 3). For salmon, MSM however had significantly less total SFA and PUFA, while other groups were the same. Mechanical separation also had no significant impact on the relative distribution of the different fatty acid groups when expressed as % of total fatty acids in MSM from herring and salmon compared with their input materials. On the other hand, herring and salmon protein isolates contained significantly (*p* < 0.05) lower absolute amounts of all the five fatty acid groups compared with both herring MSM and the backbone raw material, in line with its lower total fat content (see Table 1). The reduction in total n-3 PUFA after pH-shift processing was from 47 mg/g DW in herring backbones to 15 mg/g DW in its protein isolate, and for salmon from 17 to 11 mg n-3 PUFA/g DW. However, pH-shift processing at the same time caused a dramatic change in the relative distribution of fatty acids in the protein isolated compared with the backbone and MSM of the two species. For example, pH-shift processing increased the % PUFA in the total fatty acid pool from 24% in the herring backbones to 43% in protein isolates, and LC n-3 PUFA from 18 to 39%. In MSM, relative PUFA and LC n-3 PUFA levels were 28% and 16%, respectively. Contrary, the percentages of n-6 PUFA and MUFA were reduced from 7 and 41% in backbones to 3 and 18%, respectively, during pH-shift processing. In herring MSM, relative levels on n-6 PUFA and MUFA were 9 and 37%, respectively. The % of DHA of total fatty acids in protein isolate of herring and salmon doubled and tripled, respectively, compared with their backbone and MSM. Altogether, the relative changes in fatty acid distributions increased the n-3/n-6 ratio 4- and 2-fold for herring and salmon pH-shift produced protein isolates, respectively. This is most probably related to a relatively larger removal of storage lipids than membranes in the pH-shift process; i.e., there was a certain enrichment of phospholipids in the protein isolates, which are known to contain more of the highly unsaturated n-3 PUFA than the lipid droplets [35]. We have previously shown that the lipids recovered as an emulsion layer in the first centrifugation during pH-shift processing of herring and salmon by-products contained less phospholipids compared to oils extracted from these materials using a classic heat-based method [9]. This is probably due to the amphiphilic nature and high density of phospholipids which makes them distribute into mainly the solubilized protein fraction and/or into the insoluble sediment during the first centrifugation, while in the second centrifugation, some phospholipids remain in the aqueous supernatant. 

For cod, both processes raised the content of PUFA, n-3 PUFA and LC n-3 PUFA in the recovered protein enriched ingredients ≥ 2-fold compared with original cod backbones, with protein isolates showing significantly (*p* < 0.05) higher content of PUFA and n-3 PUFA compared with the MSM. However, pH-shift processing reduced the relative amount of n-3 PUFA and LC n-3 PUFA in the fatty acid pool of the cod protein isolate to less than half of its % in cod backbones and MSM. Contrary, the % of n-6 PUFA increased up to 4-fold compared with cod backbones and MSM. The % of EPA and DHA in the fatty acids also dramatically decreased in the cod protein isolates reaching 1.3 and 0.8%, respectively, compared with their percentage in cod backbones (6.5 and 15%, respectively) and MSM (7 and 16%, respectively), which in turn reduced the n-3/n-6 ratio in cod protein isolate down to a tenth of the ratio in cod backbone. As explained before, a visible emulsion layer is not formed during the first step when pH-shift processing cod backbones. This means that the removal of lipids from the cod input raw material takes place via precipitation into the insoluble first sediment or by remaining in the second supernatant. In agreement with this, it has earlier been shown that up to 68–75% of the input lipids could be removed when applying the pH-shift process to menhaden or krill with relatively low lipid content (15 and 24%, dw, respectively) [30], despite that no emulsion layer was formed. Both these species were ascribed a high content of phospholipids [36]. Thus, more pronounced removal of phospholipids containing a higher amount of n-3 PUFA in parallel with oxidation of the most unsaturated PUFA as EPA and DHA during the pH-shift process [9] and Wu et al., in manuscript, may have induced the changes measured in the fatty acid composition in cod protein isolate compared with its input material and MSM. 

Overall, the absolute content of the three LC n-3 PUFAs; i.e., EPA, DPA and DHA was highest in products from herring, followed by cod, and then salmon. These findings reflect the large amount of plant-based lipids in the feed for salmon [37]. The recommended daily intake of DHA and EPA is 250 mg for maintenance of cardiovascular health for children and healthy adults [38], which could be achieved by eating 32, 123 and 105 g of herring, salmon and cod MSM, respectively, equalized to 80% moisture (Supplementary Table S1). Corresponding numbers for herring, salmon and cod protein isolated on a 80% moisture basis would be 81, 140 and 96 g, respectively. Based on the general function health claims approved by EFSA (Commission Regulation (EU) No 432/2012) [39], all products would qualify for the claims related to normal function of the heart (≥40 mg EPA + DHA/100 g) as well as to normal brain function and normal vision (both with a threshold of ≥40 mg DHA/100 g product) when normalized to 80% moisture content. 

### 3.5. Vitamins 

**Figure 2:**
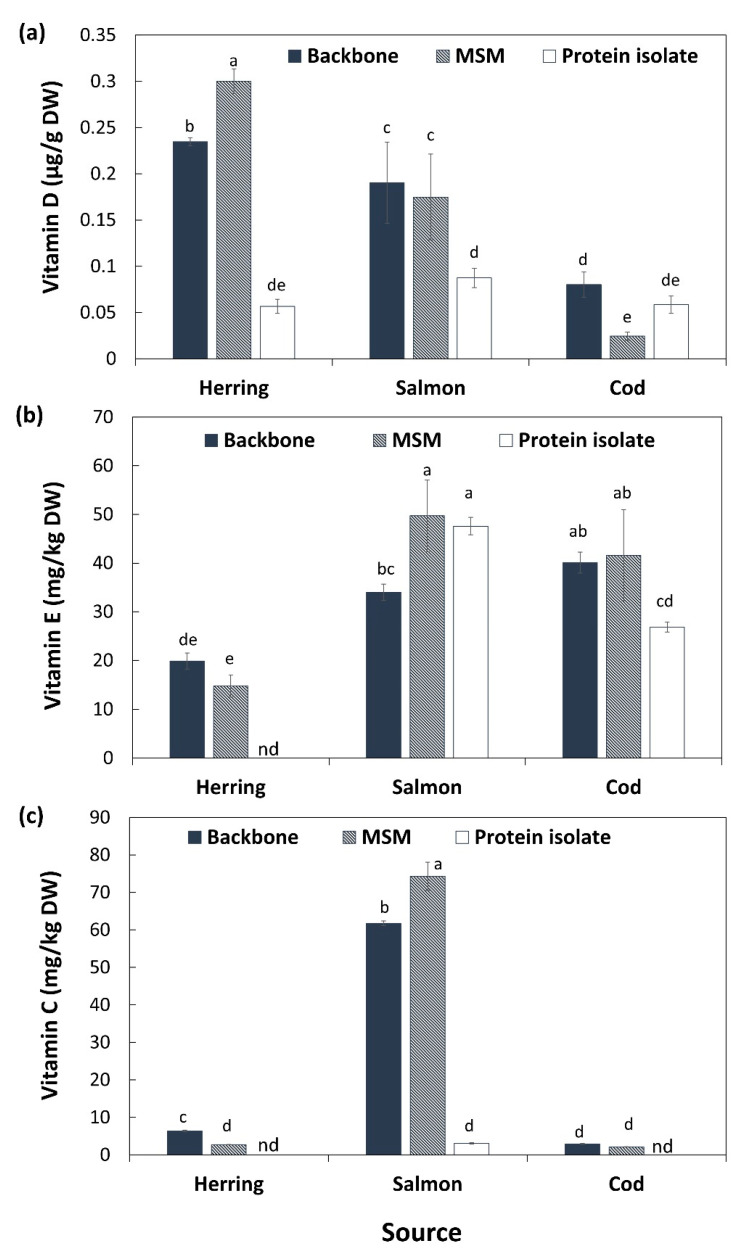
Content of vitamin D (**a**), vitamin E (**b**) and vitamin C (**c**) on dry weight (DW) basis in backbones of salmon, cod and herring as well as their mechanically separated meats (MSM) and protein isolates. Results are shown as mean ± SD (*n* = 2). nd: not detectable. Different small letters show significant differences (*p* ≤ 0.05).

Fish is a very important dietary source of the lipid soluble vitamin D, which is critical e.g., for bone health since it plays several important roles in our body as a hormone in the regulation of calcium and phosphorus metabolism [40]. Initial vitamin D content of herring backbones (0.23 µg/g DW) and salmon backbones (0.19 µg/g DW) was significantly (*p* < 0.05) higher than its content in cod backbone (0.08 µg/g DW) (Figure 2a). This shows that all three studied fish backbones can be good sources of vitamin D-rich products but that the backbones of fatty fish indeed are richer sources of this vitamin than lean fish. A large variation in vitamin D content of raw fish muscle and fishery products, ranging from 0 to 47 µg/100 g of fresh fish or product, has been reported [40,41]. Mechanical separation did not significantly (*p* > 0.05) change the content of vitamin D of salmon backbones while it significantly (*p* < 0.05) increased its content in herring MSM (0.30 µg/g DW) compared with its original backbones. pH-shift processing on the other hand caused a product with significantly (*p* < 0.05) less vitamin D for both herring (0.05 µg/g DW) and salmon (0.08 µg/g DW) compared with corresponding MSM and backbones, following the results of total fat (Table 1). Opposite, mechanical separation of cod backbones concentrated vitamin D in the MSM (0.02 µg/g DW), while pH-shift processing gave a cod protein isolate with similar levels (0.05 µg/g DW) compared with its backbone. The daily recommended intake of vitamin D in the Nordic region is 10 μg for persons < 75 years, and 20 μg > 75 years (Nordic Council of Ministers, 2014); based on EFSA, it is 5 µg in all age groups. To achieve the daily recommended intake of vitamin D of 10 μg, the daily intake for herring, salmon and cod MSM with 80% moisture (Supplementary Table S1) must be approximately 166, 286 and 2042 g, respectively, or for protein isolate it must be 882, 571, and 855 g, respectively. However, all products except the cod MSM qualified for EFSAs general function health claim related to vitamin D’s contribution to normal absorption/utilization of calcium/phosphorus, normal blood calcium levels and maintenance of normal bones, muscle function, teeth, cell division and immune system [39]; i.e., 15% of the EU RDI/100 g, i.e., 0.75 µg/100 g.

Fish products can also be good sources of the membrane-bound vitamin E, comprising four tocopherols and four tocotrienols [42]; molecules which are also important antioxidants. Cod backbones had the highest content of vitamin E (40 mg/kg DW) followed by salmon (34 mg/kg DW) and herring (19 mg/kg DW) (Figure 2b). Content of tocopherols in fish depends highly on their diet since they cannot synthesize this vitamin. The enrichment of salmon feed with tocopherols [43] can therefore explain the higher level in salmon than herring. That cod had the highest levels could be a combination of diet and the fact that this species as a lean fish has a relatively higher membrane lipid content (>80%) per amount of total lipids [35] compared to herring and salmon. That tocopherols are membrane bound was most likely a contributing reason why they responded differently to processing compared to vitamin D. Another likely reason is that they are important antioxidants. Mechanical separation of herring and cod backbones did not change the content of vitamin E in the resulting MSM. However, pH-shift processing resulted in a significantly (*p* < 0.05) lower content of vitamin E in protein isolates of both herring (<our detectable level) and cod (26 mg/kg DW) compared with their backbone and MSM. This could be related to the high amount of low molecular weight (LMW) iron and heme iron [44] measured in these samples (Table 4), stimulating free radical production which in turn can consume tocopherols during the pH-shift process. As indicated above, we have earlier documented significant lipid oxidation during the pH-shift process [45]; more so compared with mechanical separation (Wu et al., in manuscript).

In contrast with cod and herring, applying both processing technologies on salmon backbones resulted in a significantly (*p* < 0.05) higher content of vitamin E in its MSM (49 mg/g DW) and protein isolate (47 mg/g DW) compared with its backbone (35 mg/g DW). The increase in MSM would imply that bone marrow is not a significant source of vitamin E, while the increase in protein isolates is ascribed the natural abundance of astaxanthin in salmon muscle, protecting it from lipid oxidation during pH-shift processing [45], as well a relatively larger removal of lipid deposits than membranes.

Salmon backbones had a substantially higher content of vitamin C, i.e., ascorbic acid (61 mg/kg DW), compared with herring (6.3 mg/kg DW) and cod backbones (2.9 mg/kg DW) (Figure 2c). Additionally, here the results could reflect the enrichment of fish feed with vitamins; in the case of vitamin C to prevent oxidation as well as to stimulate collagen production and the immune system [46]. Mechanical separation led to significantly (*p* < 0.05) higher content of vitamin C in salmon MSM (74 mg/kg DW) compared with its backbone, while it resulted in a significantly (*p* < 0.05) lower content of vitamin C in herring MSM compared with its backbone (2.7 mg/kg DW). For cod, levels were the same in MSM and raw material. Applying pH-shift processing on the backbones of the three species resulted in a substantial reduction of vitamin C content (down to 3 mg/kg DW) in salmon protein isolate and values were below the detectable level in herring and cod protein isolates. This is most probably due to the water-soluble nature of vitamin C, and thus, that it is leached out during the pH-shift process. Additionally, occurrence of lipid oxidation can consume vitamin C, as it works as an antioxidant in synergy with tocopherol [47].

### 3.6. Content of Minerals

Both MSM and protein isolates of the three studied species had significantly (*p* < 0.05) lower content of calcium, magnesium and manganese compared with their original backbones (Table 4). Protein isolates of the three species also showed significantly (*p* < 0.05) lower content of all the named minerals compared with their MSM counterpart except calcium which was only significant for cod samples. Protein isolate of salmon had significantly lower content of selenium compared with its MSM but this was not significant for cod and herring. Results are in line with the remarkable reduction of ash content after applying mechanical separation and pH-shift processing on the backbones of the three species, which is due to the efficient removal of bones which contain high levels of these minerals. Great removal of calcium and magnesium due to very efficient removal of bone residues when using alkaline pH-shift processing has previously been seen also for trout by-products [10], gutted silvers carp (*Hypophthalmichthys molitrix*) [33] and gutted herring [31]. Further, a significantly (*p* < 0.05) lower content of calcium and magnesium was also found in MSM of beheaded rainbow trout than its fillet [28].

Content of potassium in MSM of salmon and cod was significantly (*p* < 0.05) higher than in their starting raw materials. pH-shift processing, on the other hand, led to an almost 5–10-fold lower content of potassium in the protein isolates compared with the original backbones for the three species. This is probably due to the high water solubility of potassium [48], leading to its leaching into the second supernatant formed during the pH-shift process. Similarly, a 20-fold reduction in potassium was found when the pH-shift process was applied on yellowfin tuna (*Thunnus albacares*) roe [49]. These authors confirmed that the largest removal takes place by leaching, as they found very low levels of potassium in the insoluble fraction removed after first centrifugation of the pH-shift process. 

MSM recovered from the backbone of salmon showed significantly (*p* < 0.05) lower content of zinc and copper compared with their original backbones. On the other hand, protein isolate of salmon had significantly higher content of zinc compared with salmon MSM and salmon backbone. However, pH-shift processing did not significantly (*p* > 0.05) change the content of zinc in the protein isolates of herring and cod compared with their corresponding backbones and MSM. Copper content of salmon, cod and herring protein isolates was also significantly higher than their corresponding backbones and MSM. This could be due to the high binding capacity of these two metals to proteins related to their role as cofactors in enzymes, a phenomenon which even concentrated them in salmon protein isolate. Previously, Marmon and Undeland [31] found significantly higher content of zinc and copper in protein isolates from gutted herring using the pH-shift process compared with its starting raw material. Lee et al. [49] showed that precipitation at pH 5.5 led to slightly but significantly (*p* < 0.05) higher content of zinc in protein isolates of yellowfin tuna roe compared with its starting raw material, but a significant reduction in the content of zinc was found when precipitating the proteins at pH 4.5.

Mechanical separation and pH-shift processing of salmon and cod backbones did not change the content of iron in MSM and protein isolates of compared with the original backbones, showing an equal partitioning between bones, proteins, lipids and soluble phase. However, herring protein isolates had significantly (*p* < 0.05) higher content of iron (71 mg/kg DW) compared with its MSM (52 mg/kg DW) and its original backbone (45 mg/kg DW). Previous studies have also shown an equal or higher iron content of protein isolates produced with pH-shift processing and input material. For example, the iron content of proteins isolated from gutted carp [33] and trout by-products [10] did not differ from their original input materials while it was significantly (*p* < 0.05) higher in proteins isolated from gutted herring [31] and tuna roe [49] compared with starting raw materials. 

Herring backbones had two- and four-fold higher content of heme iron (31 mg/kg DW) compared with cod (16 mg/kg DW) and salmon backbone (7 mg/kg DW). Heme iron was concentrated two-fold in the MSM and protein isolate of cod compared with its backbone and it was slightly, yet significantly (*p* < 0.05), concentrated in salmon MSM. Other products were not significantly different from their corresponding starting material. Significantly higher content of heme pigments in proteins isolated from cod by-products (head + backbone) compared with the raw material has also been reported earlier [14]. We have earlier seen that heme-iron can be removed during the pH-shift processing both by precipitation into the first sediment or by solubilization in the second supernatant, the latter accounting for the largest removal [50]. In the same study, Hb removal with the supernatant increased when recovering proteins at a Ph > or < the pI as this reduced co-precipitation of Hb with the myofibrillar proteins. In the present study we recovered proteins at the pI, which could explain the heme-iron concentrating effect for cod backbones. Another explanation could be a partitioning of heme into cellular membranes upon Hb/Mb oxidation and heme-loss [51] taking place along with lipid oxidation. More lipid hydroperoxides formed upon pH-shift processing of cod backbones than herring and salmon backbones (Wu et al., in manuscript). 

From the perspective of lipid oxidation during subsequent storage of isolates or MSM, a high heme removal is indeed ideal. However, from a nutritional point of view, presence of heme-iron is important due to its higher bioavailability compared to LMW-iron [52].

For salmon, sodium was more than twice as high in the pH-shift produced protein isolate than in corresponding backbones. There were also slight, but still significant (*p* < 0.05), sodium increases in MSM and protein isolates of cod backbones. 

Overall, the effects from the mechanical separation and pH-shift process on the mineral content of protein recovered from the fish backbones appeared to be dependent on whether the minerals are located in the bone or muscle fraction of backbones as well as their binding affinity to proteins vs. their water solubility. Minerals enriched in the bone, such as calcium and magnesium, were removed to a higher degree by the pH-shift process than the mechanical separation, while a reverse trend was seen for minerals with high binding affinity to protein e.g., zinc. Highly water-soluble minerals, e.g., potassium, were only slightly affected by mechanical separation while they were severely leached out by the pH-shift process. Fish species and composition of input materials are indeed also factors that define the content of minerals in the final protein-enriched products. 

From a health perspective, all three protein isolates contained >15% of RDI for copper when expressed on a 80% moisture basis (Supplementary Table S1), i.e., levels denoted as significant according to EFSA [39], allowing for functional health claims related to maintenance of normal connective tissues, energy-yielding metabolism, skin pigmentation and function of the immune system as well as to the protection of cells from oxidative stress. However, the EU Directive 90/496/EEC suggests 10% of RDI to be enough to denote a significant amount of a specific trace element, which applied to selenium (herring MSM), potassium (cod MSM), copper (herring MSM) and iron (herring protein isolate). The same directive also suggests that 5% of RDI can be enough to say there is a significant level, which applied to selenium in all six protein enriched products, to zinc in all three protein isolates and cod MSM, to iron for both of the cod and herring-derived products, to magnesium for cod and herring MSM, and to manganese for both types of cod products and herring protein isolate.

## 4. Conclusions

Mechanical separation resulted in higher protein yield from backbones of cod, herring and salmon compared with pH-shift processing. However, the pH-shift process produced a protein enriched product with higher purity due to more effective removal of fat from salmon and herring, and ash from all the three resources. Products recovered from backbones with both technologies were good sources of valuable nutrients such as essential amino acids, n-3 PUFA, vitamin D, copper, selenium, potassium and iron; for the latter both heme and non-heme. However, the exact content of these nutrients in the recovered protein ingredients was dependent on the type of processing technology and species. As an example, the higher purity of the pH-shift produced protein enriched product from salmon and herring led to significantly lower content of n-3 PUFA and vitamin D compared with the corresponding MSM. In case of cod, the content of these nutrients showed a reverse trend between isolate and MSM. Relative composition of fatty acids (%) was dramatically affected by pH-shift processing resulting in a higher n-3/n-6 ratio in herring and salmon protein isolates but a lower n-3/n-6 ratio in cod protein isolate compared with their MSM. Further, the pH-shift process produced protein isolates of the three resources with substantially lower content of selenium, calcium, magnesium, and potassium compared with their corresponding MSM, but higher or similar content of zinc, iron, copper and manganese. Altogether, our study showed that the type of valorization technology has a big impact on the nutrient profile of protein enriched ingredients derived from seafood side streams, an effect which varies with species. More studies on the effect of both technologies on the storage stability and product forming capacity of the recovered protein ingredients are now needed. 

## Figures and Tables

**Table 1:** Proximate composition of salmon, cod and herring backbones and their mechanically separated meat (MSM) and protein isolates. Results are shown as mean ± SD (*n* = 3).

		Moisture (%)	Protein * (% dw)	Fat * (% dw)	Ash * (% dw)
Herring	Backbone	72.91 ± 0.20 ^e^	50.82 ± 0.31 ^e^	34.22 ± 3.07 ^b^	10.24 ± 0.59 ^c^
	MSM	76.58 ± 0.51 ^d^	58.50 ± 0.74 ^d^	31.62 ± 3.56 ^b^	5.12 ± 0.30 ^d^
	Protein isolate	80.54 ± 0.08 ^ab^	85.66 ± 0.98 ^a^	7.43 ± 0.70 ^e^	2.41 ± 0.03 ^f^
Salmon	Backbone	57.95 ± 0.11 ^g^	34.60 ± 0.93 ^g^	52.03 ± 2.96 ^a^	28.38 ± 0.45 ^b^
	MSM	68.02 ± 2.43 ^f^	46.78 ± 1.5 ^f^	43.11 ± 1.32 ^b^	3.70 ± 0.53 ^e^
	Protein isolate	79.69 ± 0.00 ^b^	72.33 ± 0.52 ^c^	20.58 ± 0.24 ^d^	2.18 ± 0.04 ^f^
Cod	Backbone	77.77 ± 0.30 ^cd^	60.25 ± 1.60 ^d^	6.22 ± 0.38 ^e^	29.69 ± 0.22 ^a^
	MSM	82.92 ± 0.55 ^a^	81.07 ± 2.68 ^b^	8.85 ± 1.88 ^e^	5.49 ± 0.30 ^d^
	Protein isolate	81.01 ± 0.20 ^a^	82.85 ± 0.30 ^b^	9.29 ± 0.58 ^f^	2.51 ± 0.28 ^f^

* Protein, fat and ash content are presented based on g/100 g of dry weight (dw). Different small letters in each column shows a significant difference (*p* < 0.05).

**Table 2:** Amino acid composition (mg/g protein) of salmon, cod and herring backbones and their mechanically separated meat (MSM) and protein isolates. For comparison recommended levels of the essential amino acids (EAA) are shown to the right.

Amino Acid (mg/g Protein)	Herring			Salmon			Cod			FAO/WHO Adult (Infant) (mg/g Protein)
	Backbone	MSM	Protein Isolate	Backbone	MSM	Protein Isolate	Backbone	MSM	Protein Isolate	
Valine *	45.3 ± 1.6 ^b^	45.4 ± 1.6 ^b^	50.4 ± 1.8 ^b^	50.1.1 ± 0.9 ^b^	50.3 ± 5.6 ^b^	56.2 ± 1.3 ^a^	39.9 ± 2.7 ^c^	45.5 ± 2.2 ^b^	49.2 ± 1.4 ^b^	39(55)
Threonine *	45.1 ± 1.2 ^de^	45.7 ± 1.0 ^cde^	48.4 ± 1.8 ^bcd^	52.3 ± 2.2 ^ab^	51.0 ± 6.5 ^abc^	55.3 ± 1.0 ^a^	42.3 ± 3.0 ^e^	46.2 ± 2.0 ^cde^	48.5 ± 1.9 ^bcd^	23(31)
Isoleucine *	41.1 ± 0.8 ^d^	43.5 ± 1.3 ^cd^	48.0 ± 1.8 ^b^	48.7 ± 1.7 ^b^	49.9 ± 5.5 ^b^	55.8 ± 0.9 ^a^	39.4 ± 2.6 ^d^	46.3 ± 1.8 ^bc^	49.6 ± 1.8 ^b^	30(32)
Leucine *	41.1 ± 2.5 ^e^	50.0 ± 2.7 ^d^	61.2 ± 1.6 ^b^	52.1 ± 0.0 ^cd^	55.1 ± 4.7 ^c^	67.7 ± 1.4 ^a^	44.2 ± 2.2 ^e^	55.4 ± 1.2 ^c^	64.3 ± 1.2 ^ab^	59(66)
Lysine *	77.5 ± 0.9 ^bcd^	73.2 ± 1.9 ^cd^	77.3 ± 1.8 ^bcd^	86.0 ± 4.0 ^ab^	83.7 ± 11.1 ^ab^	88.4 ± 1.3 ^a^	68.9 ± 4.6 ^d^	76.6 ± 3.5 ^bcd^	79.8 ± 2.5 ^abc^	45(57)
Methionine *	46.9 ± 1.3 ^abc^	44.3 ± 0.7 ^bc^	44.6 ± 1.8 ^bc^	39.2 ± 7.7 ^a^	52.3 ± 2.3 ^ab^	51.2 ± 1.5 ^a^	42.1 ± 2.9 ^c^	45.2 ± 2.2 ^bc^	46.8 ± 2.2 ^abc^	17(42)
Histidine *	76.5 ± 0.5 ^ab^	68.0 ± 0.5 ^bcd^	62.0 ± 3.6 ^d^	82.4 ± 4.7 ^a^	76.9 ± 12.3 ^ab^	72.5 ± 2.03 ^abc^	63.2 ± 4.6 ^cd^	64.8 ± 4.2 ^cd^	62.8 ± 3.1 ^cd^	15(20)
Phenylalanine *	48.7 ± 2.8 ^bc^	47.8 ± 0.7 ^bc^	48.8 ± 2.8 ^bc^	55.8 ± 2.4 ^a^	53.4 ± 6.9 ^ab^	56.4 ± 1.1 ^a^	44.4 ± 3.3 ^c^	48.2 ± 2.4 ^bc^	49.9 ± 1.6 ^bc^	19(72)
Glycine	51.0 ± 0.2 ^bc^	44.3 ± 0.4 ^cde^	42.3 ± 1.8 ^e^	60.7 ± 1.8 ^a^	50.4 ± 7.1 ^bc^	49.5 ± 0.9 ^bcd^	53.2 ± 5.8 ^b^	44.4 ± 2.1 ^cde^	43.3 ± 1.8 ^de^	
Alanine	54.0 ± 0.6 ^ab^	48.5 ± 1.5 ^b^	49.9 ± 2.6 ^b^	58.6 ± 2.0 ^a^	53.8 ± 6.5 ^ab^	57.0 ± 1.7 ^a^	48.9 ± 4.5 ^b^	49.2 ± 2.1 ^b^	50.5 ± 1.2 ^b^	
Serine	58.8 ± 0.9 ^abcd^	54.4 ± 0.3 ^bcd^	52.0 ± 2.5 ^d^	65.2 ± 3.4 ^a^	60.9 ± 9.0 ^ab^	60.4 ± 1.5 ^abc^	52.8 ± 4.4 ^cd^	53.8 ± 2.8 ^bcd^	54.0 ± 2.2 ^bcd^	
Proline	48.8 ± 1.0 ^bc^	45.9 ± 1.0 ^bc^	45.8 ± 2.3 ^bc^	58.7 ± 1.7 ^a^	51.1 ± 7.0 ^bc^	52.0 ± 0.8 ^c^	49.3 ± 1.7 ^bc^	44.7 ± 2.2 ^c^	44.5 ± 2.1 ^bc^	
Aspartic acid	122.5 ± 3.0 ^d^	107.0 ± 1.1 ^bc^	98.7 ± 5.3 ^c^	129.2 ± 7.4 ^a^	120.7 ± 19.4 ^ab^	114.6 ± 3.0 ^abc^	99.5 ± 7.3 ^c^	102.3 ± 6.5 ^c^	100.4 ± 4.9 ^c^	
Glutamic acid	118.1 ± 2.8 ^ab^	104.3 ± 1.0 ^bcd^	99.3 ± 4.7 ^cd^	125.3 ± 7.0 ^a^	117.1 ± 18.4 ^ab^	113.8 ± 2.5 ^abc^	96.9 ± 7.0 ^d^	101.0 ± 5.5 ^cd^	102.0 ± 3.7 ^cd^	
Arginine	121.1 ± 3.1 ^ab^	105.9 ± 0.7 ^bcd^	97.5 ± 5.2 ^d^	128.5 ± 5.5 ^a^	120.0 ± 19.1 ^ab^	113.8 ± 3.0 ^abc^	99.4 ± 7.4 ^cd^	101.9 ± 6.4 ^cd^	99.6 ± 4.4 ^cd^	
Tyrosine	92.3 ± 2.3 ^ab^	81.3 ± 0.6 ^bcd^	74.7 ± 4.3 ^d^	98.0 ± 15.7 ^a^	91.8 ± 14.7 ^ab^	87.8 ± 2.5 ^abc^	75.5 ± 5.4 ^d^	78.4 ± 4.9 ^cd^	76.7 ± 4.0 ^cd^	
Total EAA	422.5 ± 8.16 ^cde^	418 ± 9.5 ^de^	440.8 ± 18.5 ^bcd^	479.0 ± 18.4 ^ab^	470.4 ± 59.6 ^abc^	503.6 ± 9.2 ^a^	384.5 ± 26.1 ^e^	428.3 ± 19.4 ^cde^	450.9 ± 15.8 ^bcd^	
EAA/AA	0.41 ^c^	0.41 ^c^	0.44 ^a^	0.40 ^d^	0.41 ^c^	0.43 ^a^	0.40 ^d^	0.42 ^b^	0.44 ^a^	

Results are shown as mean ± SD (*n* = 3). * Shows essential amino acids. EAA means the total content of essential amino acids. AA means the total amount of amino acids. Different small letters in each column shows significant differences (*p* < 0.05).

**Table 3:** Content of saturated (SFA), monounsaturated (MUFA) and polyunsaturated fatty acid (PUFA) in mg/g dry weight (DW) and as % of total fatty acids (data shown in parentheses) of backbones of salmon, cod and herring and their mechanically separated meats (MSM) and protein isolates.

Fatty Acids	Herring			Salmon			Cod		
	Backbone	MSM	Protein Isolate	Backbone	MSM	Protein Isolate	Backbone	MSM	Protein Isolate
∑SFA	79.25 ± 8.74 ^c^ (31.0 ± 0.37)	68.33 ± 7.84 ^c^ (29.26 ± 0.66)	11.56 ± 1.89 ^g^ (28.26 ± 2.31)	219.7 ± 13.0 ^a^ (57.2 ± 0.25)	181.3 ± 4.89 ^b^ (55.9 ± 0.09)	80.79 ± 0.66 ^c^ (57.8 ± 0.22)	16.70 ± 2.83 ^f^ (48.4 ± 4.21)	25.04 ± 1.71 ^e^ (45.8 ± 1.69)	46.30 ± 2.02 ^d^ (25.5 ± 0.05)
∑MUFA	106 ± 18.75 ^a^ (41.4 ± 4.26)	86.51 ± 9.07 ^a^ (37.0 ± 0.62)	7.30 ± 0.53 ^e^ (17.91 ± 0.13)	64.84 ± 4.78 ^b^ (16.8 ± 0.17)	53.32 ± 1.38 ^b^ (16.4 ± 0.01)	19.21 ± 0.12 ^c^ (13.7 ± 0.15)	5.87 ± 0.42 ^f^ (17.0 ± 2.52)	9.08 ± 0.00 ^d^ (16.6 ± 0.59)	59.94 ± 3.06 ^b^ (33.0 ± 0.27)
∑PUFA	60.70 ± 4.69 ^b^ (23.8 ± 3.61)	67.19 ± 3.87 ^b^ (28.8 ± 0.87)	17.47 ± 1.72 ^d^ (42.84 ± 0.21)	84.39 ± 6.64 ^a^ (21.9 ± 0.24)	67.67 ± 1.48 ^b^ (20.8 ± 0.13)	30.01 ± 0.23 ^c^ (21.47 ± 0.00)	8.60 ± 0.30 ^e^ (24.8 ± 1.00)	15.20 ± 2.40 ^d^ (27.7 ± 2.96)	33.78 ± 1.45 ^c^ (21.1 ± 0.12)
C20:5 n3 (EPA)	18.06 ± 2.05 ^a^ (7.0 ± 0.28)	15.69 ± 2.11 ^a^ (6.7 ± 0.31)	3.78 ± 0.37 ^b^ (9.26 ± 0.18)	0.28 ± 0.00 ^d^ (0.07 ± 0.00)	0.22 ± 0.00 ^e^ (0.07 ± 0.00)	0.06 ± 0.00 ^f^ (0.04 ± 0.00)	2.27 ± 0.05 ^c^ (6.5 ± 0.34)	3.03 ± 0.62 ^bc^ (7.2 ± 0.84)	3.98 ± 0.30 ^b^ (1.31 ± 0.02)
C22:5 n3 (DPA)	0.49 ± 0.37 ^cd^ (0.31 ± 0.00)	0.71 ± 0.09 ^c^ (0.30 ± 0.01)	0.09 ± 0.01 ^f^ (0.23 ± 0.02)	2.19 ± 0.20 ^a^ (0.05 ± 0.01)	1.80 ± 0.00 ^b^ (0.05 ± 0.01)	0.98 ± 0.00 ^c^ (0.7 ± 0.00)	0.16 ± 0.00 ^e^ (0.46 ± 0.03)	0.21 ± 0.02 ^d^ (0.48 ± 0.05)	0.26 ± 0.04 ^d^ (0.97 ± 0.06)
C22:6 n3 (DHA)	24.95 ± 1.78 ^a^ (9.7 ± 0.02)	23.01 ± 2.99 ^a^ (9.85 ± 0.41)	11.66 ± 0.85 ^c^ (28.6 ± 0.18)	11.05 ± 0.90 ^cd^ (2.8 ± 0.05)	9.92 ± 0.07 ^d^ (3.0 ± 0.10)	8.89 ± 0.09 ^e^ (6.3 ± 0.04)	5.21 ± 0.18 ^f^ (15.0 ± 0.60)	8.77 ± 1.13 ^de^ (16.5 ± 2.36)	9.07 ± 1.62 ^cde^ (0.76 ± 0.05)
∑LC n-3 PUFA	43.81 ± 3.85 ^a^ (17.1 ± 0.24)	39.42 ± 5.21 ^a^ (16.8 ± 0.75)	15.88 ± 1.27 ^c^ (38.1 ± 0.02)	13.53 ± 1.11 ^bc^ (3.5 ± 0.06)	11.96 ± 0.08 ^c^ (3.6 ± 0.11)	9.94 ± 0.10 ^d^ (7.1 ± 0.04)	7.65 ± 0.23 ^e^ (22.1 ± 0.98)	13.35 ± 2.27 ^bc^ (24.3 ± 3.26)	14.53 ± 0.15 ^b^ (3.0 ± 0.14)
∑n-3 PUFA	47.26 ± 3.34 ^a^ (18.0 ± 0.96)	42.82 ± 5.60 ^a^ (18.32 ± 0.24)	15.98 ± 0.85 ^c^ (39.2 ± 0.3)	17.45 ± 1.42 ^bc^ (4.5 ± 0.08)	15.03 ± 0.06 ^c^ (4.6 ± 0.13)	11.10 ± 0.09 ^d^ (7.9 ± 0.03)	7.74 ± 0.24 ^e^ (22.3 ± 0.98)	13.40 ± 2.24 ^c^ (24.51 ± 3.27)	17.73 ± 0.68 ^b^ (9.7 ± 0.13)
∑n-6 PUFA	19.56 ± 3.30 ^bc^ (7.4 ± 1.86)	22.40 ± 1.00 ^b^ (9.62 ± 0.43)	1.49 ± 0.33 ^d^ (3.64 ± 0.52)	66.63 ± 5.19 ^a^ (17.3 ± 0.16)	52.39 ± 1.34 ^a^ (16.17 ± 0.00)	18.82 ± 0.11 ^c^ (13.46 ± 0.03)	0.74 ± 0.05 ^e^ (2.14 ± 0.00)	1.48 ± 0.02 ^d^ (2.72 ± 0.11)	16.05 ± 0.77 ^c^ (8.8 ± 0.05)
n-3/n6 ratio	2.42 ± 0.71 ^b^	1.90 ± 0.16 ^b^	10.88 ± 1.64 ^a^	0.26 ± 0.00 ^f^	0.28 ± 0.00 ^f^	0.59 ± 0.00 ^e^	10.42 ± 0.41 ^a^	9.03 ± 1.57 ^a^	1.10 ± 0.00 ^c^

Results are shown as mean ± SD (*n* = 3). LC n-3 PUFA (long chain n-3 PUFA) = EPA + DPA + DHA. Different small letters in each row show a significant difference (*p* < 0.05).

**Table 4:** Mineral contents (mg/kg dry weight, DW) of salmon, cod and herring backbones, mechanically separated meat (MSM) and protein isolate. Results are shown as mean ± SD (*n* = 2).

Minerals (mg/kg DW)	Herring			Salmon			Cod		
	Backbone	MSM	Protein Isolate	Backbone	MSM	Protein Isolate	Backbone	MSM	Protein Isolate
Sodium	6435 ± 247 ^ab^	6201 ± 354 ^ab^	6749 ± 409 ^a^	2548 ± 209 ^c^	2648 ± 32 ^c^	6418 ± 307 ^ab^	5794 ± 421 ^b^	6694 ± 183 ^a^	6501 ± 133 ^a^
Calcium	7818 ± 373 ^b^	652 ± 32 ^cd^	165 ± 13 ^d^	8456 ± 783 ^b^	586 ± 41 ^cd^	204 ± 23 ^d^	9274 ± 528 ^a^	1251 ± 79 ^c^	173 ± 16 ^d^
Potassium	12,491 ± 269 ^b^	11,321 ± 803 ^bc^	1240 ± 108 ^f^	4896 ± 508 ^e^	7299 ± 940 ^d^	986 ± 18 ^f^	10,732 ± 700 ^c^	14,893 ± 231 ^a^	1277 ± 68 ^f^
Selenium	284 ± 11 ^a^	274 ± 14 ^ab^	234 ± 20 ^b^	253 ± 12 ^ab^	247 ± 15 ^ab^	178 ± 22 ^c^	281 ± 15 ^ab^	248 ± 27 ^ab^	194 ± 28 ^bc^
Zinc	31.9 ± 2.5 ^cd^	22.9 ± 1.8 ^de^	33.1 ± 5.2 c^d^	32.9 ± 3.6 ^cd^	17.7 ± 2.3 ^e^	56.8 ± 7.2 ^a^	43.9 ± 3.1 ^b^	33.9 ± 5.7 ^bc^	39.5 ± 4.8 ^bc^
Copper	5.65 ± 0.45 ^b^	5.05 ± 0.84 ^bc^	9.85 ± 1.58 ^a^	5.36 ± 0.25 ^bc^	2.60 ± 0.82 ^c^	11.11 ± 1.27 ^a^	4.64 ± 0.12 ^bc^	4.51 ± 1.38 ^bc^	10.81 ± 2.27 ^a^
Iron	45.8 ± 2.1 ^bc^	52.7 ± 5.7 ^b^	71.7 ± 7.3 ^a^	18.2 ± 0.65 ^d^	21.0 ± 1.64 ^d^	21.2 ± 2.4 ^d^	39.9 ± 7.1 ^c^	43.6 ± 3.5 ^bc^	42.9 ± 4.9 ^bc^
Heme-iron	31.90 ± 2.02 ^ab^	33.76 ± 0.23 ^a^	27.11 ± 0.28 ^b^	7.03 ± 0.39 ^e^	9.97 ± 0.17 ^d^	8.26 ± 0.001 ^de^	16.60 ± 1.48 ^c^	31.80 ± 1.60 ^ab^	29.62 ± 1.15 ^b^
Magnesium	1496 ± 34 ^b^	1286 ± 82 ^c^	55 ± 4 ^f^	927 ± 101 ^d^	631 ± 21 ^e^	75 ± 0.9 ^f^	2246 ± 83 ^a^	1183 ± 37 ^c^	70 ± 2 ^f^
Manganese	12.2 ± 1.7 ^c^	1.9 ± 0.3 ^d^	5.3 ± 0.7 ^c^	14.6 ± 1.1 ^ab^	4.2 ± 0.8 ^cd^	4.3 ± 0.9 ^cd^	15.5 ± 0.2 ^a^	5.9 ± 1.0 ^c^	5.8 ± 1.9 ^c^

Different small letters in each row show a significant difference (*p* < 0.05).

## Supplementary Materials

The following are available online at https://www.mdpi.com/article/10.3390/foods10050950/s1, Table S1: Average content of different micronutrients in 100 g of mechanically separated meat (MSM) and protein isolates of herring, salmon and cod.
