# Supplementary material for: Impact of Processing Technology on Macro- and Micronutrient Profile of Protein-Enriched Products from Fish Backbones
Source: Foods. 2021 Apr 27;10(5):950. doi: 10.3390/foods10050950 (PMC8145710; doi:10.3390/foods10050950)
Supplement: Supplementary file 1 [file foods-10-00950-s001.zip › foods-1153962-supplementary.pdf]

Supplementary Table 1, Average content of different micronutrients in 100 g of mechanically separated meat (MSM) and protein isolates of herring, salmon and cod.

Amount in 100g wet product	Herring		Salmon		Cod	
	MSM	Protein isolate	MSM	Protein isolate	MSM	Protein isolate
EPA (mg/100 g WW)	313.80	75.60	4.40	1.20	60.60	79.60
DPA (mg/100 g WW)	14.20	1.80	36.00	19.60	4.20	5.20
EPA+DHA (mg/100 g WW)	328.00	77.40	40.40	20.80	64.80	84.80
DHA (mg/100 g WW)	460.20	233.20	198.40	177.80	175.40	181.40
Vit D (μg/100 g WW)	6.00	1.13	3.50	1.75	0.49	1.17
Vit E (mg/100 g WW)	0.29	ND	0.99	0.95	0.83	0.54
Vit C (mg/100 g WW)	0.0035	0.0017	0.006	0.0011	0.0049	0.0011
Protein (g/100g DM)	11.70	17.13	9.35	14.47	16.21	16.57
Sodium (mg/100 g WW)	124.02	134.98	52.96	128.36	133.88	130.02
Calcium (mg/100 g WW)	13.04	3.30	11.72	4.08	25.02	3.46
Potassium (mg/100 g WW)	226.42	24.80	145.98	19.72	297.86	25.54
Selenium (μg/100 g WW)	5.48	4.68	4.94	3.56	4.96	3.88
Zinc (mg/100 g WW)	0.46	0.66	0.35	1.14	0.68	0.79
Copper (mg/100 g WW)	0.10	0.20	0.05	0.22	0.09	0.22
Iron (mg/100 g WW)	1.05	1.43	0.42	0.42	0.87	0.86
Heme-iron (mg/100 g WW)	0.68	0.54	0.20	0.17	0.64	0.59
Magnesium (mg/100 g WW)	25.72	1.10	12.62	1.50	23.66	1.40
Manganese (mg/100 g WW)	0.04	0.11	0.08	0.09	0.12	0.12
